# Associations between pre-operative cholesterol levels with long-term survival after colorectal cancer surgery: a nationwide propensity score–matched cohort study

**DOI:** 10.1007/s00384-024-04735-3

**Published:** 2024-10-10

**Authors:** Lea Löffler, Maliha Mashkoor, Ismail Gögenur, Mikail Gögenur

**Affiliations:** 1 Center for Surgical Science, Zealand University Hospital, Lykkebækvej 1, 4600 Køge, Denmark; 2 Department of Clinical Medicine, University of Copenhagen, Blegdamsvej 3B, 2200 Copenhagen, Denmark; 3 Danish Colorectal Cancer Group, Copenhagen, Denmark

**Keywords:** Colorectal cancer, Cholesterol, Surgery, Survival

## Abstract

**Purpose:**

Altered lipid metabolism frequently occurs in patients with solid cancers and dyslipidemia has been associated with poorer outcomes in patients with colorectal cancer. This study sought to investigate whether cholesterol levels are associated with clinical outcomes and can serve as survival predictors.

**Methods:**

We conducted a retrospective cohort study with Danish patients diagnosed with colorectal cancer who had surgery with curative intent for UICC stages I to III between 2015 and 2020. Using propensity score adjustment, we matched patients in a 1:1 ratio to examine the impact of total cholesterol (TC) > 4 mmol/L vs. ≤ 4 mmol/L within 365 days prior to surgery on overall survival (OS) and disease-free survival (DFS).

**Results:**

A total of 3443 patients were included in the study. Median follow-up time was 3.8 years. Following propensity score matching, 1572 patients were included in the main analysis. There was no statistically significant difference in OS or DFS between patients with TC > 4 mmol/L compared with TC ≤ 4 mmol/L (HR: 0.82, 95% CI, 0.65–1.03, HR: 0.87, 95% CI, 0.68–1.12, respectively.). A subgroup analysis investigating TC > 4 mmol/L as well as low-density lipoprotein (LDL) > 3 mmol/L found a significant correlation with OS (HR: 0.74, 95% CI, 0.54–0.99).

**Conclusion:**

TC levels alone were not associated with OS or DFS in patients with colorectal cancer. Interestingly, higher TC and LDL levels were linked to better overall survival, suggesting the need for further exploration of cholesterol's role in colorectal cancer.

**Trial registration:**

Not applicable.

**Supplementary Information:**

The online version contains supplementary material available at 10.1007/s00384-024-04735-3.

## Introduction

Lipids, a diverse group of biomolecules, serve as the foundational structure of biological membranes, while also functioning as signaling molecules and a source of energy [1]. One of the most significant metabolic changes in cancer is the modification of lipid metabolism. Cancer cells exhibit increased synthesis or uptake of lipids, which can fuel their rapid growth and promote the formation of cancer cells [2].

Dyslipidemia as well as other sequelae related to metabolic syndrome have been associated with poorer outcomes in patients with breast and colorectal cancer (CRC) [3–5], suggesting dyslipidemia to be a risk factor for other cancers as well. Thus, an increasing body of research links dysregulated cholesterol homeostasis to the pathobiology of solid cancers, as cholesterol is imperative to cell proliferation and growth, being an essential constituent for the membrane of the cell [6]. Moreover, cholesterol metabolites can affect both the innate and adaptive immune system, through the induction of CD8 + T-cell exhaustion when in the tumor microenvironment [7] and have both pro- and anti-inflammatory properties, possibly contributing to the state of chronic inflammation [8].

In both cancer and viral diseases, dyslipidemia may thus contribute to immune dysfunction, exacerbating inflammation [9]. Notably, the overlap in immune response mechanisms, such as CD8 + T-cell exhaustion in the tumor microenvironment and potential immune dysregulation during viral infections, highlights the intricate interplay between dysregulated lipid metabolism and compromised immunity in diverse disease contexts.

The hypothesis in the present study was that pre-operative elevated lipid levels worsen clinical outcome with regard to overall survival (OS) and disease-free survival (DFS). The aim of this research was to investigate different circulating lipid levels to see if these could potentially be used as predictors of survival in patients with CRC.

## Methods

This retrospective propensity score–adjusted study was based on a cohort of Danish patients diagnosed with colorectal cancer who received surgery with curative intent for stages one to three of the Union of International Cancer Control (UICC) between 2015 and 2020. The study follows the “Strengthening the Reporting of Observational Studies in Epidemiology” (STROBE) guidelines [10].

### Data sources

The data sources utilized were from the Danish Colorectal Cancer Group (DCCG), the Danish National Patient Registry (DNPR), the Danish Registry of Laboratory Results for Research (RLRR), and the Danish National Prescription Registry (NPR). The national DCCG database has existed since 2001 and includes all patients with a Danish social security number over the age of 18 with primary CRC, either diagnosed or treated surgically at a public Danish hospital [11]. The DNPR was founded in 1977 and offers national, extensive longitudinal registration of administrative and clinical data, including information on treatments, examination procedures, and diagnoses in- and outpatient facilities [12]. All individual biochemistry results since 2008, sourced from both private healthcare providers and public medical institutions, are recorded in the RLRR [13]. Lastly, the NPR has been maintaining national data on all dispensed prescription drugs since 1994, proving to be an excellent resource for evaluating the effects of drug use in large populations under routine care, with potential for long-term follow-up [14].

Patient data from the four sources were integrated into a unified Observational Medical Outcomes Partnership Common Data Model (OMOP CDM) using open-source analytics tools developed by the Observational Health Data Sciences and Informatics (OHDSI) community. The CDM aligns with OMOP standards and was used for the purposes of data retrieval and subsequent analysis [15].

### Study population

The study encompassed all individuals aged 18 or above who received surgical treatment with curative intent for UICC stages one to three colorectal cancer between the years 2015 and 2020. We excluded patients with metastases and emergency surgery as well as those with non-curative surgery (as determined during the operation). Patients in the target cohort included those with total cholesterol (TC) above 4 mmol/L at any time up to 365 days pre-operatively, whereas patients in the comparator cohort had measurements of 4 mmol/L or less. This cut-off value was chosen in accordance with the recommended TC levels outlined by the European Society of Cardiology (ESC) and the European Atherosclerosis Society (EAS) [16].

If a subject appeared in both the target and comparator cohorts, only their first occurrence in only one of the cohorts was retained. Sample size calculations were not performed.

### Primary and secondary outcomes

The primary outcome of the study was to investigate how cholesterol measurements up to 1 year prior to surgery were associated with OS in patients with CRC. OS was defined as the interval from operation until either death or the conclusion of the follow-up period (May 2020). The secondary outcome was DFS, in which the time at risk was specified as 180 days post-surgery until either cancer relapse, death, or conclusion of the follow-up period. DFS was determined using the validated algorithm developed by Lash et al. [17] [18].

An unadjusted analysis where target and comparator cohorts were not matched was also performed to examine the unmitigated effects of the exposure variable on the primary and secondary outcomes. To evaluate the precision of the study design, five negative control scenarios were integrated (Supplementary Table 1) [19].

### Subgroup analyses

The majority of the subgroup analyses conducted in our study were pre-specified in a protocol uploaded to the Open Science Framework (OSF) in February 2023 (available at https://osf.io/vsaht/). This protocol outlines the primary and secondary outcomes, along with the planned subgroup analyses. The rationale for these pre-specified subgroup analyses was to explore potential variations in treatment effects across different subpopulations, which we deemed crucial for understanding the generalizability and applicability of the study findings.

These pre-specified analyses looked at TC > 4 mmol/L as opposed to ≤ 4 mmol/L in subgroups for both the primary and secondary outcome, including within patients classified as ASA I-II, UICC I-II, females or males only, and colon and rectal tumor as well as a group looking at age > 70 years.

Furthermore, we implemented four exploratory analyses to investigate the relationship between various lipid marker cut-offs and the primary and secondary outcomes, serving as sensitivity analyses to assess the robustness of the findings. These analyses included the following: total cholesterol (TC) > 4 mmol/L and low-density lipoprotein (LDL) > 3 mmol/L; TC > 4 mmol/L, LDL > 3 mmol/L, and high-density lipoprotein (HDL) > 1.2 mmol/L; TC > 5 mmol/L; and TC > 4 mmol/L combined with exposure to statins 1 year prior to surgery.

This last subgroup was of interest as patients with total cholesterol levels at or near the threshold of TC = 4 are less likely to be prescribed statins in accordance with established guidelines in Denmark [20]. Treatment with statins in Denmark is typically initiated based on various risk factors such as age and cardiovascular disease risk factors rather than solely on total cholesterol levels.

The specific lipid cut-offs were chosen as they are consistent with Danish desired values for TC, LDL, and HDL (< 5 mmol/L, < 3 mmol/L [21] and > 1.2 mmol/L [20], respectively).

Lastly, a subgroup analysis was performed to compare the patient group with cholesterol measurements as opposed to those that did not have measurements of cholesterol to assess potential health-seeking bias.

Two post hoc sensitivity analyses were performed. In the first sensitivity analysis, any duplicate subjects were removed, while in the second, only the latest occurrence was retained instead of the first occurrence.

### Data security

Approval for this study was granted by the Danish Data Protection Agency under the approval number REG-102–2020. The data utilized in the study underwent anonymization and cloud-based secure storage on the platform Computerome 2.0, where all analytical procedures were performed. Source data cannot be shared according to Danish legislation, due to containing sensitive personal information.

### Statistical analyses

Propensity score (PS) models were fitted for the main and subgroup analyses using the Cyclops package for R [22]. Cohorts were matched in a 1:1 ratio using the corresponding propensity scores that represent the likelihood of a patient in the comparator cohort to have total cholesterol measurements above 4 mmol/L compared to 4 mmol/L or less. The cohorts were matched using a greedy nearest neighbor method. No replacement of controls was utilized in matching.

These propensity scores were calculated using all available data such as demographic data, drug exposure, and medical observations as well as previous medical procedures and prior medical history. These covariates were matched with a look-back period of any time prior to the surgery as well as 2 years prior. Covariates related to cholesterol levels as well as post-operative covariates were not included (Supplementary Table 2). Quantitative biochemical measurements contributed to the propensity score calculation through the following methods: Patients were matched based on if a given analysis had been made, the numerical values obtained for each analysis, and whether the biochemical results fell within or exceeded the normal range.

For instances where records were missing in the database, e.g., an absent diagnosis, we considered this to mean the patient does not have the diagnosis.

In this study, a maximum caliper width of 0.2 standard deviation units on the logit scale was used to match the patients, adhering to recommended practices and serving as the recognized optimal threshold for ensuring effective patient matching [23]. The PS model was then evaluated through visual inspection of Kernel density plots of the propensity scores and scatter plots to assess covariate balance [24]. Additionally, the standardized mean difference (SMD) of covariates was calculated to ensure comparability between treatment groups. The pre-specified threshold for SMD difference was 0.1, as recommended [25].

A Cox proportional hazards model was used to estimate both OS as well as DFS. The results are presented as hazard ratios (HR) with 95% confidence intervals (CI). HR values greater than 1 indicate an increased risk of death or disease progression among individuals with TC > 4 mmol/L. Conversely, HR values less than 1 suggest a reduced risk. Statistical significance was determined using a threshold of *p*-value less than 0.05. Proportional Hazards Assumption check was done to assess whether hazard ratios are constant throughout the study period. For this assessment, graphical methods like log–log survival curves and Schoenfeld residuals were used.

The open-source tool ATLAS, developed by the OHDSI community, was used to construct the target and comparator cohorts while all analyses were performed in R-studio (version 4.2.0) using the HADES package (v1.12.0) [26].

## Results

**Fig. 1:**
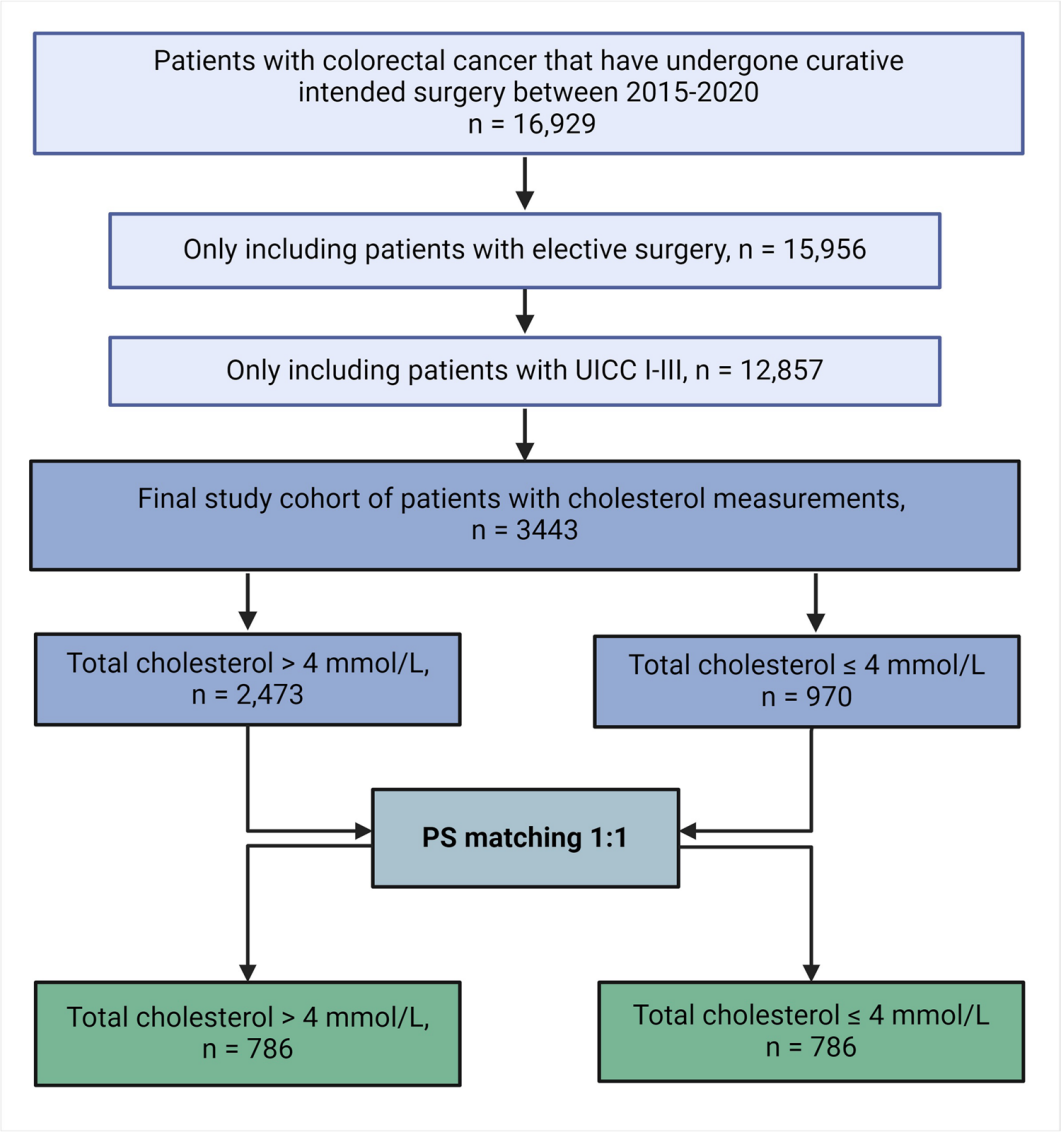
Flowchart of the study population

We identified 12,857 patients that met the inclusion criteria in the study period 2015–2020. A total of 2473 patients were identified in the target group, having TC measurements of > 4 mmol/L, and 970 patients in the comparator group with ≤ 4 mmol/L 365 days pre-operatively (Fig. 1).

After applying the criteria of first exposure and propensity score matching, a total of 786 patients remained in each cohort; thus, 1572 patients were included in the final analysis. The relatively low number of patients included in the final analysis can be attributed to the fact that cholesterol blood samples are not routinely conducted as part of standard care in Denmark, both at the general practitioner level unless specific risk factors are present, nor prior to colorectal cancer surgery.

The median follow-up time in the analysis of overall survival was 3.8 years (interquartile range [IQR] 2.3–5.5 years) and 3.48 years for disease-free survival (IQR 1.98–5.25 years).

### Patient characteristics

Prior to propensity score matching, the patients in the group with TC measurements > 4 mmol/L were very similar to the patients in the comparator group with TC measurements ≤ 4 mmol/L in terms of cancer stage, performance status, body mass index, and comorbidities. We found a high SMD in the age and gender variable before matching, which is reduced below the threshold after propensity score matching (see Table 1). The characteristics noted in our study mirror the ones of patients with CRC within the broader population, as outlined in the annual report by the DCCG from 2015 and 2020, respectively [27, 28]. However, as per the 2015 report, it is important to note that only 77.9% of the patients who underwent surgery that year had a precise UICC stage reported, likely due to variations in procedures and guidelines at the time.

**Table 1:** Selected patient variables before and after matching are presented. Percentages are shown in parentheses. The total number for tumor localization may vary from the cohort size due to synchronous cancer sites within the cohort. Abbreviations used: *ASA* American Society of Anesthesiologists, *BMI* body mass index, *ECOG* Eastern Cooperative Oncology Group, *SMD* standardized mean difference, *UICC* Union for International Cancer Control

	Before propensity score matching			After propensity score matching		
Variables	TC > 4 mmol/L	TC ≤ 4 mmol/L	SMD	TC > 4 mmol/L	TC ≤ 4 mmol/L	SMD
*N*	2473	970	-	786	786	-
Age						
Age, mean	70.8	72.7	-0.20	72.3	72.5	-0.02
Sex						
Female	1099 (44.4)	271 (27.9)	0.35	270 (34.4)	247 (31.4)	0.06
Male	1375 (55.6)	699 (72.1)	-0.35	516 (65.6)	539 (68.6)	-0.06
HMG-CoA reductase inhibitor treatment	877 (35.5)	742 (76.5)	-0.91	587 (74.7)	558 (71.1)	0.08
BMI, kg*m^−2^						
≤ 18.5	41 (1.7)	12 (1.2)	0.04	14 (1.8)	8 (1.0)	0.06
> 18.5 to ≤ 25.0	853 (34.5)	341 (35.1)	-0.01	272 (34.6)	274 (34.8)	-0.01
> 25.0 to ≤ 30.0	932 (37.7)	376 (38.8)	-0.02	303 (38.6)	307 (39.1)	-0.01
> 30.0 to ≤ 35.0	378 (15.3)	141 (14.5)	0.02	108 (13.7)	111 (14.1)	-0.01
> 35.0	186 (7.5)	64 (6.6)	0.04	59 (7.5)	55 (7.0)	0.02
Missing	83 (3.4)	36 (3.7)	-	30 (3.8)	31 (3.9)	-
Charlson comorbidity index						
0	1302 (52.6)	488 (50.3)	0.05	413 (52.6)	394 (50.1)	0.05
1	495(20.0)	213 (22.0)	-0.05	153 (19.5)	174 (22.1)	-0.07
2	355 (14.4)	137 (14.1)	0.01	132 (16.8)	113 (14.4)	0.07
3	321 (13.0)	132 (13.6)	-0.02	88 (11.2)	105 (13.3)	-0.07
WHO Performance Status						
0	1139 (46.0)	442 (45.6)	0.01	363 (46.2)	359 (45.7)	0.01
1	548 (22.1)	205 (21.1)	0.02	163 (20.7)	165(21.0)	-0.01
2	135 (5.5)	65 (6.7)	-0.05	51 (6.5)	51 (6.5)	0.00
3	27 (1.1)	15 (1.5)	-0.04	10 (1.3)	12 (1.5)	-0.02
4	< 6 (0.0)	NA	NA	< 6 (0.0)	NA	NA
Missing	624 (25.2)	243 (25.0)	-	199 (25.3)	199 (25.3)	-
ASA						
1	335 (13.5)	130 (13.4)	0.00	104 (13.2)	103 (13.1)	0.00
2	1493 (60.3)	580 (59.8)	0.01	478 (60.8)	473 (60.2)	0.01
3	591 (23.9)	244 (25.2)	-0.03	188 (23.9)	196 (24.9)	-0.02
4	31 (1.3)	9 (0.9)	0.03	7 (0.9)	7 (0.9)	0.00
5	< 6 (0.0)	NA	NA	NA	NA	NA
Missing	23 (0.9)	7 (0.7)	-	9 (1.1)	7 (0.9)	-
Pathological UICC stage						
I	551 (22.3)	250 (25.8)	-0.08	176 (22.4)	202 (25.7)	-0.08
II	953 (38.5)	354 (36.5)	0.04	295 (37.5)	294 (37.4)	0.00
III	806 (32.6)	308 (31.8)	0.02	261 (33.2)	240 (30.5)	0.06
Missing	163 (6.6)	58 (6.0)	-	54 (6.9)	50 (6.4)	-
Tumor localization						
Rectum	1195 (48.3)	487 (50.2)	-0.04	381 (48.5)	399 (50.9)	-0.05
Sigmoid colon	408 (16.5)	171 (17.6)	-0.03	135 (17.2)	136 (17.3)	-0.00
Descending colon and splenic flexure	127 (5.1)	45 (4.6)	0.02	45 (5.7)	33 (4.2)	0.07
Transverse colon	169 (6.8)	62 (6.4)	0.02	56 (7.1)	52 (6.6)	0.02
Ascending colon and hepatic flexure	320 (12.9)	117 (12.1)	0.03	98 (12.5)	96 (12.2)	0.01
Cecum	261 (10.5)	93 (9.6)	0.03	74 (9.4)	74 (9.4)	0.00
Synchronous cancer	7	< 6	-	< 6	< 6	-

### Propensity score matching

**Fig. 2:**
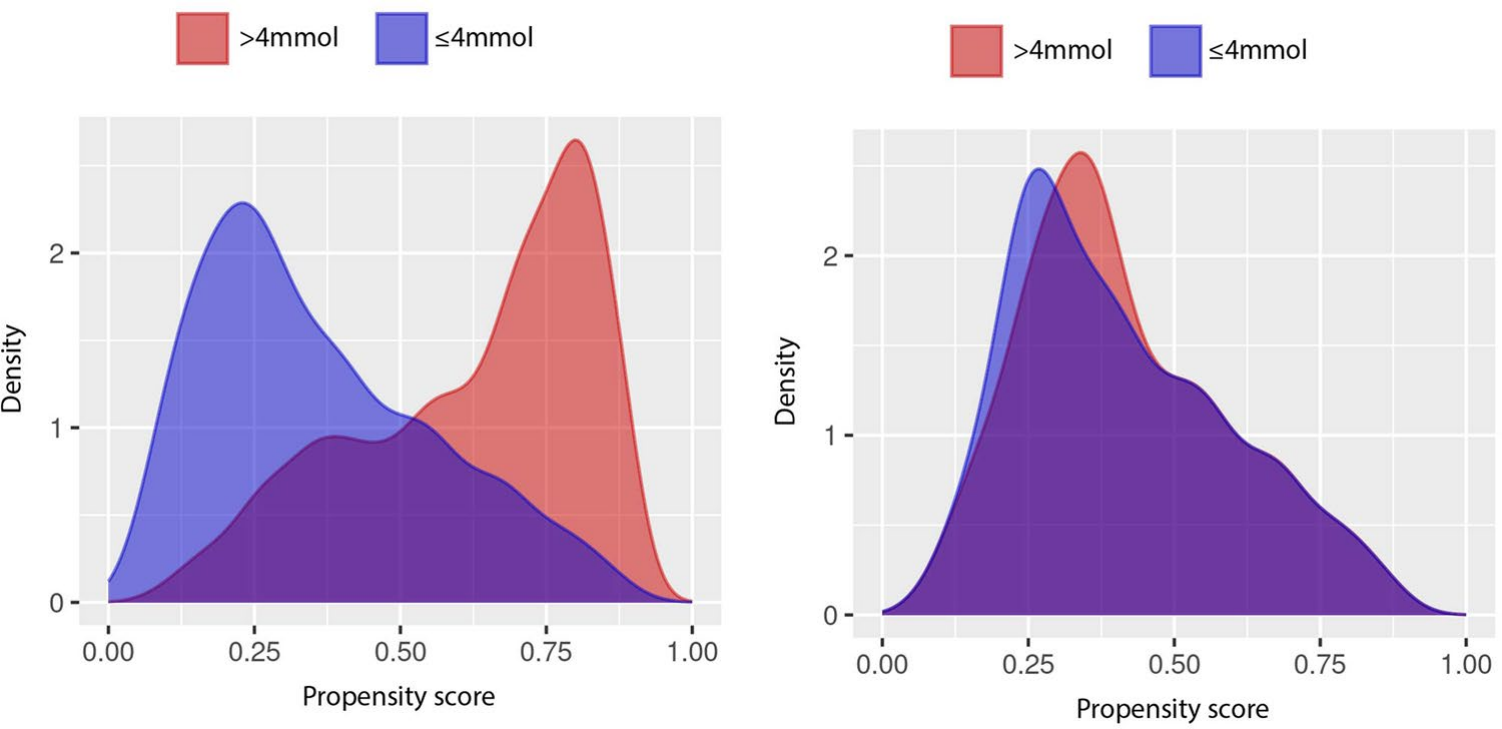
Dispersion of patient propensity score in the primary cohort before (left) and following propensity score matching (right)

The density plots of the propensity scores pre- and post-matching are illustrated in Fig. 2. Furthermore, the scatterplot showing the SMD pre- and post-matching reveals that 1.3% of the covariates (549/42,190) had a SMD beneath − 0.1 or over 0.1 after matching (Supplementary Fig. 2). A total of 123 variables remained from the total pool of variables in computing the propensity score.

### Overall survival

In an unadjusted analysis before propensity score matching, there was a statistically significant difference in OS between the > 4 mmol/L and ≤ 4 mmol/L group (HR: 0.69, 95% CI, 0.60–0.79; *p* < 0.001). The 5-year summary survival statistics also indicate a statistically significant difference between the two groups. The target group (> 4 mmol/L) had a survival rate of 76.83% (95% CI, 0.75–0.79), whereas the survival rate in the group with TC 4 ≤ mmol/L was 66.33% (95% CI, 0.63–0.70).

**Fig. 3:**
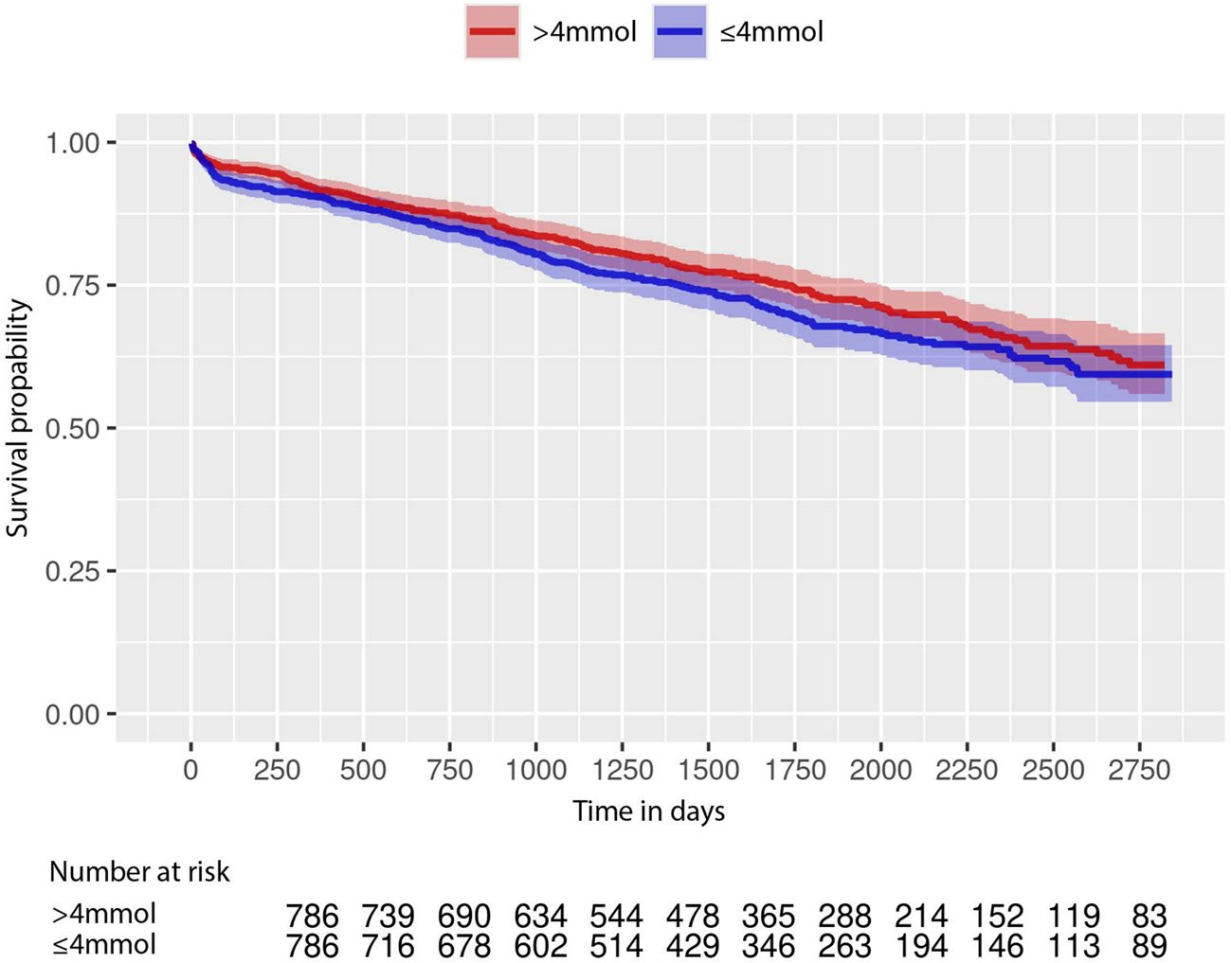
Survival outcomes in colorectal cancer patients after surgery in the adjusted primary analysis. Presented are the Kaplan–Meier curves comparing patients with TC measurements > 4 mmol/L 365 days prior to surgery (red) versus TC measurements ≤ 4 mmol/L (blue)

The association of improved OS was not evident in the adjusted main analysis of the propensity score–matched groups (HR: 0.82, 95% CI, 0.65–1.03; *p* = 0.09) (Fig. 3).

In our analysis, both the log–log plot of survival curves and the assessment of Schoenfeld residuals indicated no significant violations of the proportional hazards assumption, supporting the validity of the hazard ratio estimates presented in this study.

The first subgroup analysis found a statistically significant association between TC > 4 and LDL > 3 mmol/L vs. ≤ 4 mmol/L TC and ≤ 3 mmol/L LDL and OS (HR: 0.74, 95% CI, 0.54–0.99; *p* = 0.048).

**Fig. 4:**
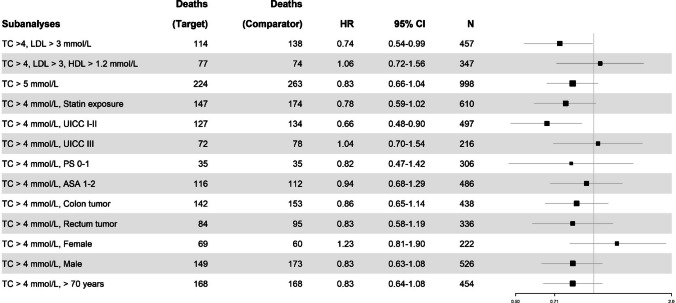
Forest plot depicting all subanalyses examining overall survival. The patient count (*N*) pertains to the number in one study arm. Results are displayed solely for the target group, not the comparator group which assessed TC ≤ 4 mmol/L across respective subgroups. The size of the black square representing the hazard ratio (HR) varies according to the number of patients included in each specific subanalysis

None of the other lipid subgroup analyses investigating overall survival showed any statistically significant results, nor did the subgroup analysis investigating TC > 4 mmol/L as well as being exposed to statins (see Fig. 4).

We found a statistically significant association with OS among patients classified as UICC I-II (HR: 0.66, 95% CI, 0.48–0.90; *p* = 0.01), with no significant association in patients classified as UICC III (HR: 1.04, 95% CI: 0.70–1.54; *p* = 0.85). Additional subgroup analyses included patients classified as WHO performance score 0–1 (HR: 0.82, 95% CI: 0.47–1.42; *p* = 0.48) and ASA I-II (HR: 0.94, 95% CI: 0.68–1.29; *p* = 0.70).

In the subgroup analysis of patients with colon tumor, there was no significant association with OS (HR: 0.86, 95% CI: 0.65–1.14, *p* = 0.29), nor for patients with rectal tumors (HR: 0.83, 95% CI: 0.58–1.19; *p* = 0.31).

There was no significant association with OS in only female patients (HR: 1.23, 95% CI: 0.81–1.90; *p* = 0.34), or male patients only (HR: 0.83, 95% CI: 0.63–1.08; *p* = 0.18).

Additionally, age was assessed as a factor, with a subgroup analysis of patients over 70 years old resulting in an HR of 0.83 (95% CI: 0.64–1.08; *p* = 0.16) (Fig. 4).

Finally, we investigated whether having a TC measurement vs. no TC measurement in our database was associated with OS, where we found no statistically significant association, although a trend was evident (HR: 0.86, 95% CI, 0.72–1.01; *p* = 0.08).

When investigating whether removing all duplicate subjects in the cohorts, 628 patients in each cohort remained, with no statistically significant association with OS (HR: 0.95, 95%CI, 0.74–1.21). Including the latest exposure instead of the first exposure of a TC level above or equal to or below 4 resulted in 743 patients in each cohort, with no statistically significant association with OS (HR: 0.95, 95%CI, 0.75–1.19).

### Disease-free survival

**Fig. 5:**
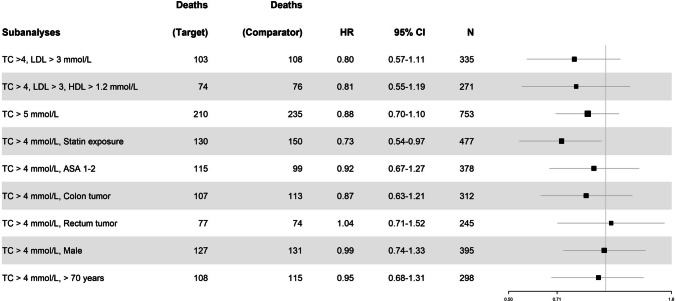
Forest plot depicting all subanalyses examining disease-free survival. The patient count (*N*) pertains to the number in one study arm. Results are displayed solely for the target group, not the comparator group which assessed TC ≤ 4 mmol/L across respective subgroups. The size of the black square representing the hazard ratio (HR) varies according to the number of patients included in each specific subanalysis

The unadjusted analysis that was performed of the groups before matching showed a statistically significant difference in DFS between the patients with TC measurements of > 4 mmol/L 365 days prior to surgery versus the group with measurements ≤ 4 mmol/L (HR: 0.81, 95% CI, 0.69–0.94; *p* = 0.01). The main adjusted analysis did not show a significant association between TC > 4 mmol/L with DFS (HR: 0.87, 95% CI, 0.68–1.12; *p* = 0.27). The subgroup analysis examining TC > 4 mmol/L in combination with exposure to statins 1 year prior to surgery showed a significant association (HR: 0.73, 95% CI: 0.54–0.97; *p* = 0.04). None of the other subgroup analyses looking at lipid levels or subdividing the cohorts with regard to ASA, tumor location, sex, or age were significant (Fig. 5).

The number of subanalyses exploring the secondary outcome, DFS, is smaller in comparison to the primary outcome, OS. This variance primarily stems from the constrained pool of patients available for specific subgroups in the secondary outcome analysis due to the distinct criteria in the validated recurrence algorithm developed by Lash et al. [18].

The subgroup analysis comparing those who have gotten their total cholesterol measured as opposed to no measurement taken within 1 year prior to surgery demonstrated no significant difference between the two groups (HR: 0.99, 95% CI, 0.83–1.19; *p* = 0.91).

## Discussion and conclusion

In this study, we explored the potential impact of pre-operative elevated lipid levels on the clinical outcomes OS and DFS, in patients with stage one to three CRC that underwent operation with curative intent between the years 2015 and 2020.

The main analysis, after adjusting for propensity scores, did not demonstrate a significant association between pre-operative TC levels above 4 mmol/L and OS in patients with CRC. Similarly, no significant association was found between elevated TC levels and DFS. However, in the first subgroup analysis, focusing on patients with both TC above 4 mmol/L and LDL above 3 mmol/L, a significantly higher OS was observed in the target group compared to the control group. No significant associations were found in the other subgroup analyses, apart from improved OS in patients with UICC I-II and TC > 4 mmol/L as well as improved DFS in patients with exposure to statins as well as TC > 4 mmol/L. Interestingly, we found a statistically significant association between improved OS and DFS in patients with TC > 4 mmol/L in the unadjusted analysis for both outcomes, highlighting the potential direct impact of cholesterol levels on outcomes before accounting for confounding factors.

These findings suggest that while pre-operative TC levels alone may not be strong prognostic markers of survival in patients with CRC, there might be an interaction between TC and LDL levels that is associated with OS. Additionally, an observed benefit on DFS may be linked to statin use.

Our findings contribute to the broader understanding of lipid metabolism in cancer, suggesting a potential paradoxical association between higher cholesterol levels and overall survival.

Lipids, including cholesterol, are essential components of cellular membranes and play critical roles in cell signaling, energy storage, and structural integrity [29]. Dysregulated lipid metabolism is a hallmark of cancer, with numerous studies demonstrating that cancer cells often exhibit altered lipid profiles to support their rapid growth and proliferation. This is also the case in studies investigating colorectal cancer, with blood cholesterol levels decreasing significantly in patients and showing a negative correlation with higher tumor or polyp grades compared to healthy individuals [30, 31]. Thus, one possible explanation is that the rapid growth and proliferation of cancer cells could deplete cholesterol reserves as these cells utilize cholesterol and other lipids to sustain their heightened metabolic demands. This depletion may contribute to the development of cachexia, a syndrome characterized by severe weight loss and muscle wasting, which is commonly observed in advanced cancer patients [32]. As a result, lower cholesterol levels might reflect a more aggressive tumor metabolism and poorer overall nutritional status, potentially leading to worse clinical outcomes. Conversely, higher cholesterol levels might indicate a more stable lipid reserve and better nutritional status, which could contribute to improved survival in cancer patients. A study conducted to investigate the metabolic changes that occur during cancer cachexia progression finds that both cholesterol and LDL, but not triglycerides or HDL, were significantly reduced in cachectic patients [33]. This underlines previous findings as well [34]. However, this hypothesis warrants further investigation and consideration in future studies, especially as the inconsistent findings on hypercholesterolemia and cancer suggest that their relationship is not simply binary but may be influenced by other factors, such as the tissue origin of the cancer or variations in daily cholesterol intake, which could serve as epigenetic regulators of cancer progression [35]. Furthermore, it is important to acknowledge that the data used in this study only encompasses patients having undergone surgery with curative intent up until May 2020; thus, part of the follow-up period for patients undergoing treatment for colorectal cancer in 2020 may have overlapped with the COVID-19 pandemic.

Emerging evidence suggests that lipid disturbances during COVID-19 might also have contributed to disease severity and complications [9, 36, 37]. Dyslipidemia could potentially worsen the immune response and exacerbate the inflammatory processes associated with severe COVID-19 cases. Given this emerging evidence, it would be intriguing to investigate in future research whether the pandemic has impacted the observed relationship between lipid metabolism and clinical outcomes in colorectal cancer patients. The confluence of dyslipidemia, immune dysfunction, and inflammation, observed in both cancer and viral diseases like COVID-19, underscores the relevance of understanding lipid metabolism in diverse disease contexts.

This study’s primary strengths lie in its extensive use of variables for the propensity score analysis, incorporating a total of 42,190 variables. This comprehensive approach enables the computing of nuanced propensity scores, effectively creating a real-world approximation of the ideal randomized controlled trial (RCT) [38]. Utilizing data from national Danish registries offers another significant advantage due to its consistent and standardized national data collection process. This uniformity ensures minimal selection and attrition bias as there would only be loss to follow-up if patients leave the country. Health-seeking bias has been accounted for in the analysis examining patients who have had their lipids measured versus the ones who have not. Furthermore, using the open-source tools provided by the OHDSI community provides a framework for observational health studies that ensure standardized analytics through the use of templates. This improves reproducibility and transparency while ensuring consistency in how data is computed.

However, while the PS matching controls for measured confounders, many covariates that would be valuable to consider when computing the propensity scores are not included in the CDM, such as health habits or socioeconomic status, with a risk of residual bias remaining. Regarding pre-analytical conditions, it is essential to note that information regarding patient fasting status prior to LDL analysis was not available. As outlined in the literature [39], fasting before sample collection is recommended for certain analyses, including LDL measurement. However, the lack of data on preceding fasting necessitates cautious interpretation of our results, as fasting status can significantly influence LDL levels. It was also evident that excluding all duplicate subjects or using only the latest occurrence of a subject yielded an association with OS that was not similar to our main approach of using the first occurrence of a subject. Using the first occurrence of a subject may be seen as a surrogate for longer exposure to a high or low TC level. Future studies could explore using a longer look-back period and maybe the mean TC level over this period, instead of only a single occurrence.

These limitations introduce inherent biases and constraints in data collection. Therefore, further studies with larger sample sizes and diverse patient populations are necessary to confirm our findings and elucidate the underlying mechanisms explaining the unexpected association between higher cholesterol levels and longer overall survival in colorectal cancer.

In conclusion, this paper explores the question of cholesterol’s impact on survival in surgery-treated patients with colorectal cancer, suggesting that, contrary to expectations, elevated total cholesterol levels alone were not associated with improved overall survival, but elevated total cholesterol and low-density lipoprotein levels were. This finding challenges established knowledge and calls for further research to elucidate the complex interactions between cholesterol metabolism, nutritional status, and cancer outcomes. Understanding these mechanisms holds promise for refining risk assessment strategies and potentially influencing patient care and outcomes.

## Supplementary Information

Below is the link to the electronic supplementary material.

Supplementary file1 (DOCX 62 KB)

## Data Availability

No datasets were generated or analysed during the current study.
