# Supplementary material for: Associations between pre-operative cholesterol levels with long-term survival after colorectal cancer surgery: a nationwide propensity score–matched cohort study
Source: Int J Colorectal Dis. 2024 Oct 10;39(1):159. doi: 10.1007/s00384-024-04735-3 (PMC11467112; doi:10.1007/s00384-024-04735-3)

**Appendices**

*Supplementary Table 1 – Negative controls, all analyses*

| **ID** | **Description** | **Domain** |
| --- | --- | --- |
| 194133 | Low back pain | Condition |
| 201620 | Kidney stone | Condition |
| 4344500 | Impingement syndrome of shoulder region | Condition |
| 4308093 | Dupuytren’s disease of palm | Condition |
| 380094 | Carpal tunnel syndrome | Condition |

*Supplementary Table 2 – Variables that were excluded in the propensity score matching analysis*

| **ID** | **Description** | **Domain** |
| --- | --- | --- |
| 3025839 | Triglyceride [Moles/volume] in Serum or Plasma | Measurement |
| 437827 | Pure hypercholesterolemia | Condition |
| 4308837 | Postoperative wound-infection-superficial | Condition |
| 4308542 | Postoperative wound-infection-deep | Condition |
| 4237450 | Postoperative wound infection | Condition |
| 4052011 | Postoperative wound abscess | Condition |
| 4059737 | Postoperative urinary tract infection | Condition |
| 2147483252 | Postoperative surgical complication | Condition |
| 4265769 | Postoperative subphrenic abscess | Condition |
| 438485 | Postoperative state | Condition |
| 200618 | Postoperative shock | Condition |
| 44782822 | Postoperative sepsis | Condition |
| 4170452 | Postoperative renal failure | Condition |
| 36717564 | Postoperative procedure | Procedure |
| 4200891 | Postoperative pneumonia | Condition |
| 4118656 | Postoperative period | Observation |
| 4056824 | Postoperative monitoring | Observation |
| 2147483246 | Postoperative medical complication | Condition |
| 4088114 | Postoperative intra-abdominal abscess | Condition |
| 4340367 | Postoperative intestinal obstruction | Condition |
| 437474 | Postoperative infection | Condition |
| 4340373 | Postoperative ileus | Condition |
| 4308536 | Postoperative hypovolemic shock | Condition |
| 137820 | Postoperative hypothyroidism | Condition |
| 4002836 | Postoperative hemorrhage | Condition |
| 4308835 | Postoperative hematoma formation | Condition |
| 4167232 | Postoperative fistula | Condition |
| 4086668 | Postoperative fever | Condition |
| 4309333 | Postoperative deep vein thrombosis | Condition |
| 4300243 | Postoperative complication | Condition |
| 4203846 | Postoperative aspiration pneumonia | Condition |
| 4134563 | Postoperative anesthesia care unit | Observation |
| 2147483250 | Postoperative anastomotic leak grade | Measurement |
| 2147362764 | Plv(spec.) – Cholesterol+ester; stofk. = ? mmol/L | Measurement |
| 436003 | Persistent postoperative fistula | Condition |
| 2147483503 | Patient fulfils criteria for recurrence eligibility | Observation |
| 2147483502 | Patient fulfils criteria for readmission eligibility | Observation |
| 2147364101 | P(fPt) – Cholesterol+ester; stofk. = ? mmol/L | Measurement |
| 2147358210 | P(fPt) – Cholesterol+ester/Cholesterol+ester, I HDL; stofratio = ? | Measurement |
| 2147357827 | P(fPt) – Cholesterol+ester, I VLDL; stofk. = ? mmol/L | Measurement |
| 2147359098 | P(fPt) – Cholesterol+ester, I LDL; stofk. = ? mmol/L | Measurement |
| 2147358084 | P(fPt) – Cholesterol+ester, I HDL; stofk. = ? mmol/L | Measurement |
| 2147352905 | P – Cholesterol+ester; subst.c = ? mmol/L | Measurement |
| 2147364099 | P – Cholesterol+ester/Cholesterol+ester, I HDL; stofration = ? | Measurement |
| 2147352908 | P – Cholesterol+ester, in VLDL; subst.c = ? mmol/L | Measurement |
| 2147352907 | P – Cholesterol+ester, in LDL; subst.c = ? mmol/L | Measurement |
| 2147352906 | P – Cholesterol+ester, in HDL; subst.c = ? mmol/L | Measurement |
| 2147352026 | P – Cholesterol+ester, i LDL; stofk. (DSKB 2017) = ? mmol/L | Measurement |
| 2147370218 | P – Cholesterol+ester(non HDL); subst.c = ? mmol/L | Measurement |
| 2147483490 | Not referred to postoperative oncological treatment due to patient death | Condition |
| 4215995 | Low cholesterol-low saturated fat diet | Observation |
| 2147297515 | Hypercholesterolæmi med endogen hyperglycæmi | Metadata |
| 2147346005 | HYPERCHOLESTEROLAEMIA NON SPECIFICATA | Metadata |
| 2147345979 | HYPERCHOLESTEROLAEMIA FAMILIA. MORBUS MULLER_HARBITZ | Metadata |
| 4134862 | Familial hypercholesterolemia | Condition |
| 197318 | Cholesterolosis of gallbladder | Condition |
| 3001318 | Cholesterol.total/Cholesterol in HDL [Percentile] | Measurement |
| 3011163 | Cholesterol.total/Cholesterol in HDL [Mass Ratio] in Serum or Plasma | Measurement |
| 4232581 | Cholesterol retinal embolus | Condition |
| 3022487 | Cholesterol in VLDL [Moles/volume] in Serum or Plasma | Measurement |
| 3001308 | Cholesterol in LDL [Moles/volume] in Serum or Plasma | Measurement |
| 3023602 | Cholesterol in HDL [Moles/volume] in Serum or Plasma | Measurement |
| 40758961 | Cholesterol esters [Moles/volume] in Serum or Plasma | Measurement |
| 40758961 | Cholesterol esters[Moles/volume] in Serum or Plasma | Measurement |
| 3019900 | Cholesterol [Moles/volume] in Serum or Plasma | Measurement |
| 3041918 | Cholesterol [Moles/volume] in Pleural fluid | Measurement |
| 40757502 | Cholesterol [Moles/volume] in Peritoneal fluid | Measurement |
| 2147357961 | Asc – Cholesterol+ester; stofk. = ? mmol/L | Measurement |
| 40759157 | Apolipoprotein B/Cholesterol in LDL [Mass Ratio] in Serum or Plasma | Measurement |

*Supplementary Figure 1 – Standard Mean Difference after propensity score matching, overall survival main analysis. The SMD value of each covariate before and after matching is represented by a blue dot, main analysis. The covariates with the SMDs furthest from -0.1 or 0.1 were the use of agents for dermatitis excluding corticosteroids, condition occurrence of intraocular hemorrhage or nail damage any time before surgery as well as two years before surgery. The discrepancy between the covariates not meeting the specified threshold was observed in a limited number of patients, ranging from 10 to 40 individuals.*


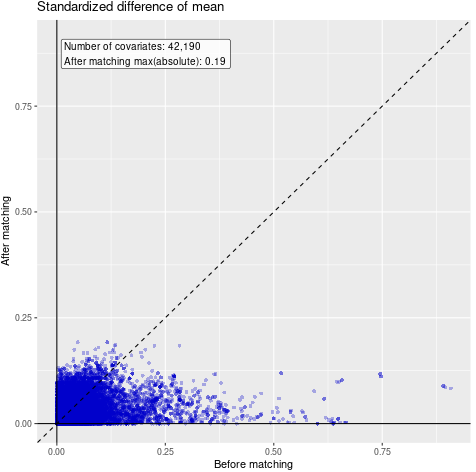

Supplement: Supplementary file 1 — Supplementary file1 (DOCX 62 KB) [file 384_2024_4735_MOESM1_ESM.docx]
